# HIV and Syphilis Infection Among Men Who Have Sex with Men — Bangkok, Thailand, 2005–2011

**Published:** 2013-06-28

**Authors:** Jintanat Ananworanich, Anupong Chitwarakorn, Wipas Wimonsate, Anchalee Varangrat, Supaporn Chaikummao, Anuwat Sriporn, Jaray Tongtoyai, Philip Mock, Wichuda Sukwicha, Wannee Chonwattana, Pikunchai Luechai, Marcel Curlin, Janet McNicholl, Timothy Holtz, Frits van Griensven

**Affiliations:** South East Asia Research Collaboration with Hawaii (SEARCH) Thailand, Thai Red Cross AIDS Research Center, Bangkok; Dept of Disease Control, Ministry of Public Health, Nonthaburi; Thailand Ministry of Public Health–U.S. CDC Collaboration, Nonthaburi, Thailand

Although efforts to control the heterosexual human immunodeficiency virus (HIV) epidemic in Thailand had shown success by the late 1990s (1), HIV continued to spread in other risk groups, including men who have sex with men (MSM). In 2003, the Thailand Ministry of Public Health–U.S. CDC Collaboration (TUC) started surveillance among MSM in Bangkok, finding an HIV prevalence of 17.3% (2). By 2005, HIV prevalence in this group had risen to 28.3% and has since stabilized at around 30% (2,3). To obtain additional information about HIV and sexually transmitted infection (STI) prevalence and incidence in a clinic-based population of MSM, TUC, in collaboration with the Thai Red Cross AIDS Research Center, analyzed data collected at the Silom Community Clinic (SCC), an HIV and STI testing center targeting MSM. This report describes trends in HIV and syphilis prevalence and incidence seen among SCC MSM clients during 2005–2011. At first clinic visit, the prevalence of HIV infection among 4,762 clients was 28.3% and of syphilis (all stages) was 9.8%. Among those returning for HIV or syphilis testing before the end of 2011, the incidence of HIV infection was 6.3 per 100 person-years (PY) and 3.6 per 100 PY for syphilis. These results show ongoing epidemics of HIV and syphilis infection in MSM in Bangkok, underscoring the urgent need for preventive interventions to reduce the spread of HIV and STI in this population.

The SCC is located in a central Bangkok hospital close to a large number of MSM entertainment venues and was founded by TUC in 2005. It supports an environment and staff receptive to the health and concerns of the MSM community; HIV and STI testing services are free, rapid, anonymous, and confidential. The long-term goal of the SCC is to build a durable relationship with the MSM community and to identify safe, effective, and affordable HIV prevention methods for MSM.

At first visit, all SCC attendees are offered HIV voluntary counseling and testing* and evaluation for primary, secondary, and latent syphilis.† Other procedures offered include testing for immunity to hepatitis A and B,§ hepatitis B vaccination (if eligible), and evaluation for the presence of gonococcal and nongonococcal urethral, pharyngeal, or rectal infections; genital ulcers; and warts. All testing, vaccination, and STI treatments are provided free of charge. Since July 2009, HIV-negative clients have been offered nucleic-acid amplification testing for acute HIV infection. Reactive nucleic-acid amplification testing in HIV antibody-negative persons is evidence of acute HIV infection, a phase of infection during which viral replication and infectiousness are high (4). All HIV-positive SCC attendees are offered a CD4+ T lymphocyte count¶ to assess their eligibility for antiretroviral therapy (ART)** and antimicrobial treatment. Thailand offers free public health care to all its citizens, and HIV ART was included in 2006 (5). HIV and syphilis prevalence were calculated at the first SCC visit during 2005–2011 when this testing was performed. HIV and syphilis incidence were computed among those who tested negative for HIV or syphilis at first visit, who returned for testing later. Times between the date of the first negative test and the midpoint between the dates of the last negative and first positive HIV or syphilis test were used to calculate PY of follow-up time. Trends in HIV prevalence and incidence were evaluated for statistical significance using chi-square and Poisson exact tests.

During the 2005–2011 period, 4,762 MSM clients made a total of 15,219 visits to the SCC (Table). Most (60.9%) clients were aged <30 years, 90.8% were Thai, 7.2% were non-Asian, and 2.0% were other Asian. Almost all (91.8%) resided in the Bangkok metropolitan area. Less than half (42.7%) reported a history of previous HIV testing. The yearly number of clients increased from 221 in 2005 to 1,135 in 2011, and the yearly number of visits increased from 439 to 4,220 during the same period. Prevalence of HIV at first visit during 2005–2011 was 28.3% and of syphilis 9.8%. HIV incidence was 6.3 per 100 PY and of syphilis was 3.6 per 100 PY of follow-up. Of the 2,736 HIV-negative specimens evaluated by nucleic-acid amplification testing since July 2009, 15 tested positive for acute HIV infection (prevalence: 0.55%). Among the 1,243 who tested HIV-positive, 41.9% had a CD4+ count of ≤350 cells/*μ*L, 29.1% had a count of >350 and ≤500 cells/*μ*L, and 29.0% had a count of >500 cells/*μ*L. Although HIV prevalence was significantly higher among older attendees (29.5% among those aged ≥30 years versus 22.8% for those aged ≤21 years), HIV incidence was significantly higher in the younger age group (12.2 per 100 PY for those aged ≤21 years versus 3.2 per 100 PY for those aged ≥30 years) (Table). From 2005 to 2011, significant increases occurred in the annual prevalence of HIV (from 24.6% to 29.4%) and syphilis (from 5.0% to 12.5%). The incidence of HIV also increased significantly from 2005–2006 to 2011, from 2.8 per 100 PY to 7.9 per 100 PY, as did the incidence of syphilis, from 0.0 per 100 PY to 7.1 per 100 PY (Table).

## Editorial Note

These data show ongoing and increasing epidemics of HIV and syphilis infection among MSM in Bangkok. While reemerging and increasing HIV and syphilis epidemics have been reported among MSM in industrialized countries (6), these data provide evidence for the existence of similar trends among MSM in a less developed country. Of particular concern are the higher HIV incidence (12.2 per 100 PY) found in MSM aged 15–21 years and the uniform syphilis incidence (3.6 per 100 PY) across all age categories.

At the first visit to the SCC, less than half of all clients reported a previous HIV test. Knowledge of HIV status has been shown to be associated with increased preventive behavior, particularly among those testing HIV-positive (7). In addition, knowledge of HIV status is necessary for linkage to care, treatment, and prevention services, including timely access to ART. A recent clinical trial of daily oral ART preexposure prophylaxis to prevent HIV infection in MSM demonstrated a 44% reduction in new HIV infections (8). In a trial of early ART of the HIV-infected partner in serodiscordant heterosexual couples, HIV transmission to the uninfected partner decreased by 96% (9). In the context of these recent advances in HIV prevention, increased HIV testing and linkage to ART in clinic-based settings such as the SCC might play an important role in reducing HIV transmission among MSM.

The findings in this report are subject to at least two limitations. First, SCC clients self-selected for HIV and STI testing, and thus might not be representative of the Bangkok MSM community. Second, seeking testing and retesting are likely associated with increased levels of risk, which might artificially bias estimates of HIV and syphilis prevalence and incidence upwards. If this bias is consistent over time, it might not affect the validity of the trend analysis, showing increases in HIV and syphilis prevalence and incidence over time.

What is already known on this topic?

Continuing, increasing, and reemerging epidemics of human immunodeficiency virus (HIV) and other sexually transmitted infections (STIs) have been reported among men who have sex with men (MSM) in the United States and elsewhere in the industrialized world. Limited information is available about the prevalence and incidence of HIV and STI infection among MSM in lower-income and lower-middle income countries.

What is added by this report?

Among MSM followed at a sexual health clinic in Bangkok, Thailand, during 2005–2011, the prevalence of HIV infection was 28.3% and of all stages of syphilis was 9.8%. Among those returning for HIV or syphilis testing before the end of 2011, the incidence of HIV infection was 6.3 per 100 person-years and of syphilis was 3.6 per 100 person-years.

What are the implications for public health practice?

The ongoing epidemics of HIV and syphilis infection in Bangkok MSM underscores the need for innovative and increased efforts to reduce their spread in this population.

The increasing HIV prevalence and incidence in Bangkok MSM are likely the result of unprotected anal intercourse, the predominant route of HIV transmission among MSM. In this situation, combined implementation of evidence-based HIV prevention interventions to reach the MSM population in Bangkok might be able to reduce HIV transmission. This could include increased acceptance of HIV testing and access to ART for earlier treatment and preexposure prophylaxis in combination with motivational programs that reduce unprotected anal intercourse and increase the use of condoms, along with lubricants that do not weaken latex, during anal intercourse.

## Figures and Tables

**TABLE:** HIV and syphilis prevalence and incidence among men who have sex with men, by age group and calendar year — Silom Community Clinic, Bangkok, Thailand, 2005–2011

Characteristic	Clients		Visits		Prevalence				Incidence			
	
					HIV		Syphilis*		HIV		Syphilis*	
					
	No.	(%)	No.	(%)	Proportion	(%)	Proportion	(%)	Rate	(per 100 PY)	Rate	(per 100 PY)
**Totals**	**4,762**	**(100)**	**15,219**	**(100)**	**1,243/4,398**	**(28.3)**	**424/4,324**	**(9.8)**	**109/1,733**	**(6.3)**	**75/2,106**	**(3.6)**
**Age group (yrs)** †												
15–21	662	(13.9)	2,222	(14.6)	148/650	(22.8)	66/635	(10.4)	25/205	(12.2)	10/242	(4.1)
22–29	2,238	(47.0)	7,046	(46.3)	603/2,078	(29.0)	182/2,041	(8.9)	61/798	(7.6)	40/1,003	(4.0)
≥30	1,862	(39.1)	5,951	(39.1)	492/1,670	(29.5)	176/1,648	(10.7)	23/730	(3.2)	25/861	(2.9)
Median (range)	7	(14–80)	—		28	(17–80)	28	(15–73)	25	(16–76)	28	(20–57)
P-value§	—		—		0.007		0.43		<0.001		0.2	
**Calendar year**												
2005	221	(4.6)	439	(2.9)	51/207	(24.6)	10/200	(5.0)	0/30	(0.0)	0/61	(0.0)
2006	554	(11.6)	1,509	(9.9)	118/514	(23.0)	16/505	(3.2)	5/147	(3.4)	0/253	(0.0)
2007	506	(10.6)	1,332	(8.7)	114/451	(25.3)	27/440	(6.1)	8/221	(3.6)	3/191	(1.6)
2008	653	(13.7)	1,842	(12.1)	162/609	(26.6)	27/611	(4.4)	14/284	(4.9)	1/312	(0.3)
2009	748	(15.7)	2,582	(17.0)	191/696	(27.4)	63/707	(8.9)	28/375	(7.5)	19/467	(4.1)
2010	945	(19.8)	3,295	(21.7)	306/898	(34.1)	157/867	(18.1)	31/384	(8.1)	30/510	(5.9)
2011	1,135	(23.8)	4,220	(27.7)	301/1,023	(29.4)	124/994	(12.5)	23/292	(7.9)	22/312	(7.1)
P-value§	<0.001		<0.001		<0.001		<0.001		<0.002		<0.001	

**Abbreviations:** HIV = human immunodeficiency virus; PY = person years.

* Includes primary, secondary, and latent syphilis.

† Age at time of prevalent (first test) and incident (subsequent test) infection.

§ Poisson exact test was used to evaluate trends in HIV and syphilis incidence; chi-square test for trend was used elsewhere.

## Footnotes

* Alere Determine HIV 1/2 (Alere Medical Co., Ltd., Chiba, Japan), Double Check II HIV 1&2 (Orgenics Ltd., Yavne, Israel) (after February 2011 replaced by SD Bioline HIV-1/2 3.0 [Standard Diagnostics Inc., Kyonggi-do, Republic of Korea]), and Capillus HIV-1/HIV-2 (Trinity Biotech PLC, Bray, Ireland) (after November 2008 replaced by Core HIV-1&2 [Core Diagnostics, Birmingham, United Kingdom]).

† Rapid plasma reagin test, confirmed by immunochromatography (Alere Determine Syphilis TP, Alere Medical Co., Ltd., Chiba, Japan).

§ Antibody against hepatitis A virus by enzyme-linked immunoassay (Murex anti-HAV [total]) and antibody against hepatitis B virus surface antigen (Murex anti-HBs, Murex Biotech Ltd., Dartford, United Kingdom).

¶ EasyCD4 System (Guava Technologies Inc., Hayward, California).

** Currently ≤350 cells/*μ*L in Thailand.
